# Analysis of shade-matching ability in dental students: a comparative study under clinical and correcting light conditions

**DOI:** 10.1186/s12909-024-05146-2

**Published:** 2024-02-22

**Authors:** Rizwan Jouhar, Muhammad Adeel Ahmed, Artak Heboyan, Muhammad Faheemuddin, Seyed Ali Mosaddad, Naseer Ahmed

**Affiliations:** 1 Department of Restorative Dental Sciences, College of Dentistry, King Faisal University, Al-Ahsa, 31982 Saudi Arabia; 2 Department of Prosthodontics, School of Dentistry, Tehran University of Medical Sciences, North Karegar St, Tehran, Iran; 3 Department of Research Analytics, Saveetha Dental College and Hospitals, Saveetha Institute of Medical and Technical Sciences, Saveetha University, Chennai, 600 077 India; 4 Department of Prosthodontics, Faculty of Stomatology, Yerevan State Medical University after Mkhitar Heratsi, Str. Koryun 2, Yerevan, 0025 Armenia; 5 Department of Prosthodontics and Implantology, College of Dentistry, King Faisal University, Al-Ahsa, 31982 Saudi Arabia; 6 Student Research Committee, School of Dentistry, Shiraz University of Medical Sciences, Shiraz, Iran; 7 Department of Prosthodontics, Altamash Institute of Dental Medicine, Karachi, 75500 Pakistan

**Keywords:** Shade matching, Aesthetic dentistry, Dental student, Education, Light source, Spectrophotometer

## Abstract

**Background:**

Selecting the ideal tooth shade is essential to the success of aesthetic dental restorations. Students’ cognitive abilities are involved in the multifaceted and intricate process of shade matching. Hence, the present study aimed to assess and compare the shade-matching ability of undergraduate dental students in various years of dental education under clinical and correcting light.

**Methods:**

This comparative cross-sectional study was instigated amongst male 4th, 5th, and 6th-year students of the dental complex of King Faisal University, Kingdom of Saudi Arabia. A total of 72 male dental students assessed the shade under clinical (fluorescent light) and correcting light (handheld Dental Base Light) by using VITA Classical shade guides. Statistical analysis was done using SPSS version 23 (Armonk, NY, USA). The Chi-square test was used to evaluate the association between correct and incorrect shade matching under correcting and clinical light.

**Results:**

Out of 72 male students, 22(30.6%) were from the 4th year, 26(36.1%) were from the 5th year, and 24(33.3%) were from the 6th year, with a mean age of 22.92 ± 1.01 years. The majority of the 6th-year students selected shade of anterior tooth # 11 correctly under clinical and correcting light, and 3(12.5%) students selected incorrectly under clinical and correcting light, with a statistically significant association among them (*p* = 0.004). As far as the shade selection of the posterior tooth is concerned, a statistically significant difference was observed under clinical light among all clinical students (*p* = 0.008).

**Conclusion:**

The clinical performance of dental students in shade matching improved with advancing years of dental education. Additionally, the shade matching ability of all groups of dental students was superior under correcting light compared to conditions under clinical light.

## Background

Achieving precise dental shade matching is a essential part of aesthetic dentistry, influenced by various factors, including the light source, recipients, background, tooth structure, and shape [1–3]. In the pursuit of objectivity, instrumental shade measurements using spectrophotometers, colorimeters, and spectroradiometers are recommended in conjunction with visual approaches [4–6]. While shade-matching devices aid medical professionals, they are not without limitations, prompting the reliance on commercial shade guides for the finalization of the process [7]. Nevertheless, these guides often fall short in adequately representing the full spectrum of tooth colors [8, 9].

Shade-matching proficiency is intricately linked to age, experience, and congenital color vision deficiencies [10–12]. The influence of professional expertise, gained through clinical experiences, is evident among dental professionals regularly undertaking restorative procedures, although some studies present conflicting views on the significance of experience [13–15].

The choice of light source plays a critical role in shade matching for artificial teeth [16]. Dentistry utilizes three primary light sources: operatory light, natural daylight, and fluorescent lights [17]. Optimal conditions for dental shade selection involve light with a temperature between 5500 and 6500 K and a Color Rendering Index (CRI) exceeding 90 [17].

Efforts to reduce the impact of ambient lighting on dental shade matching have led to the recommendation of color-corrected lighting tubes and handheld light-correcting devices [18]. The limitations of older fluorescent tubes have prompted the introduction of a new generation of more explanatory and adjustable light-correcting gadgets, with several studies attesting to their proficiency in achieving accurate color-matching results [19].

Recent research has investigated into the effectiveness of combining digital recording devices with color-correcting instruments, as well as the comparative efficacy of different light sources in dental color matching [20–23]. However, scientific evidence on the variations in visual shade matching across different types of devices remains scarce.

This study seeks to address this gap by comparing the accuracy of visual shade selection under two light sources—clinical light and correcting light sources—among male clinical students in their 4th, 5th, and 6th years. Through this exploration, we aim to contribute valuable insights into the clinical dentistry, which will help dental shade selection, and enhance best practices in aesthetic dentistry.

## Methods

This comparative cross-sectional study was conducted among male clinical dental students of different training levels at the dental complex of King Faisal University, Kingdom of Saudi Arabia, after receiving ethical approval from the Research Ethics Committee of King Faisal University (KFU-REC-2023-NOV-ETHICS1639). This study included 72 male dental students in their fourth, fifth, and sixth years of study who demonstrated normal color vision by passing the computer-based Ishihara Colour Blindness Test (24 Plate version) [24]. Students with color vision deficiencies were not included in this study. Since no student had been identified as colorblind, there was no disqualification. Every student signed a formal permission form and got accurate information about the study guidelines before enrolling.

### Patient selection and initial shade matching

The convenience sampling method was employed to choose the 72 patients sample for 72 dental students. The primary investigator chose the patient who came in for the restoration treatment and consented to be chosen for the study’s shade matching. The teeth numbered 11 and 36 were selected for shade matching. Cases that lacked 11 or 36, had prior restoration or had inherent discoloration were excluded. The chief investigator used spectrophotometers, such as Vita Easy Shade® V, to choose the initial tooth shade.

### Dental operatory selection

For shade selection, similar dental operatories were selected to maintain the clinical light condition. The color temperature of the dental operatory light was measured by using the smartphone application Light Spectrum Pro EVO having 92 to 98% accuracy (AM Power Software, Via Località Passignano, 17 04025 Lenola (LT), Italia), and the temperature range found was 3400°K ± 150°K.

### Shade selection by students

**Fig. 1:**
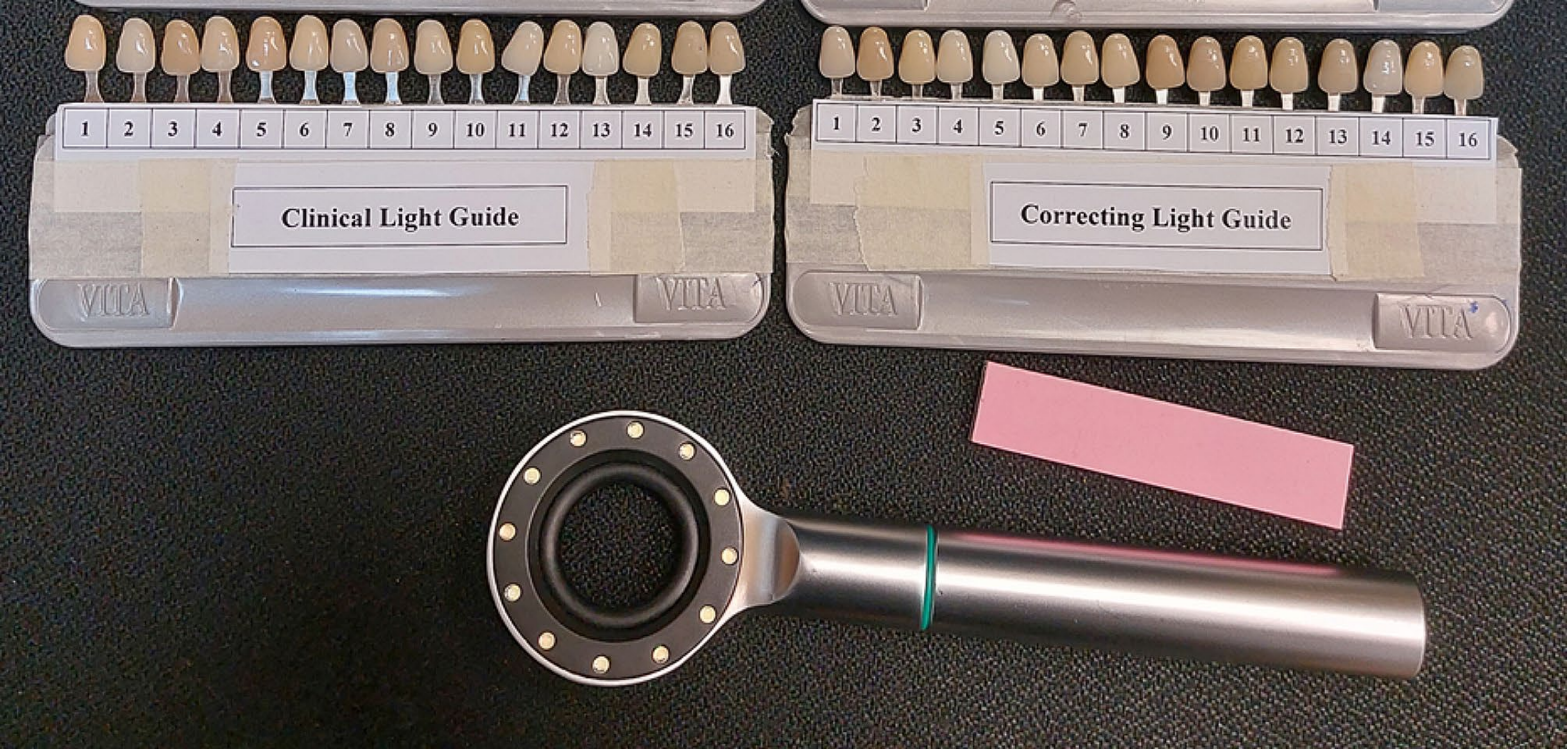
Specified shade guide for clinical and correcting light

The shade-matching process by students was carried out using two VITA Classical shade guides. Shade tabs in both shade guides was randomly aligned and the recognition of shade tabs was covered and assigned an identification code 1, 2, 3, 4, 5, and so on until 16. The principal investigator recorded the shade tab coding in both shade guides. One shade tab was labeled as a clinical light shade guide and the other one as a correcting light shade guide as shown in Fig. 1.

Students were advised to match a single shade under clinical light with a specified shade guide for the anterior tooth (#11) and posterior tooth (#36). The duration for choosing a shade was restricted to three minutes, as longer times increase the likelihood of inaccuracy. Shade-matching under the corrective light was carried out after 10-minute intervals using a handheld Dental Base Light (Tri-Shade, Zhengzhou, China) with a designated shade guide. The Dental Base Light has twelve embedded LEDs. Based on the corrective light, the optimum color temperature for shade selection was 5500°K.

All the shade matching was done in the daylight between 10:30 A.M. and 1:30 P.M. under clinical and corrective light. The number of the chosen shade tabs was recorded, and the accurate matches were computed following the comparison of the chosen items (shade tabs hiding the identifying code) with a VITA Easy Shade V shade selection.

### Statistical analysis

The data was statistically analyzed using SPSS version 25.0 (IBM Corp., Armonk, NY, USA). Categorical variables, for instance, gender and the student’s training level, were expressed as frequencies and percentages, whereas continuous variables, such as the patient’s and student’s age, were documented as mean ± SD. A Chi-square test was applied to evaluate the association between correct and incorrect shade selection of anterior and posterior teeth under clinical and correcting light with clinical students. A *p*-value < 0.05 was reflected as statistically significant.

## Results

A total of 72 male clinical students participated in this study, with a mean age of 22.92 ± 1.01 years. Among the study participants, 22(30.6%) were from the 4th year, 26(36.1%) were from the 5th year, and 24(33.3%) were from the 6th year. The mean age of the patients was 30.53 ± 10.45 years, with 45(62.5%) males and 27(37.5%) females. None of the students withdraw from this study after participation, as shown in Table 1.

**Table 1:** Demographic characteristics of male dental students (*n* = 72)

Variable		Mean ± SD n(%)
Student’s Age (years)		22.92 ± 1.01
Patient’s Age (years)		30.53 ± 10.45
Patient’s Gender	Male	45(62.5%)
	Female	27(37.5%)
Students training level	4th year	22(30.6%)
	5th year	26(36.1%)
	6th year	24**(**33.3%)

A comparison of shade selection of anterior tooth # 11 under clinical and correcting light by 4th year students revealed that 8(36.36%) students selected the shade correctly under clinical and correcting light, although it was statistically insignificant (*p* = 0.157). Around 11(42.3%) 5th year students selected the shade correctly under clinical and correcting light, and 8(30.76%) and 3(11.53%) students selected the shade incorrectly under clinical and correcting light, respectively, with an insignificant difference observed among them (*p* = 0.495). Furthermore, the majority of the 6th year students selected shade correctly under clinical and correcting light, and 3(12.5%) students selected incorrectly under clinical and correcting light, with a statistically significant association among them (*p* = 0.004), as shown in Table 2.

**Table 2:** The association of shade selection of tooth # 11 under clinical and correcting light with respect to the training level of students

Variables			Shade Selection under Correcting light (Tooth # 11)			
			Correct (n%)	Incorrect (n%)	Total (n%)	***P***-value
4th year	Shade Selection under Clinical light (Tooth # 11)	Correct	8(36.36%)	1(4.54%)	9(40.9%)	0.157
		Incorrect	8(36.36%)	5(22.72%)	13(59.1%)	
		Total	16(72.7%)	6(27.3%)	22(100.0%)	
5th year	Shade Selection under Clinical light (Tooth # 11)	Correct	11(42.3%)	3(11.53%)	14(53.8%)	0.495
		Incorrect	8(30.76%)	4(15.38%)	12(46.15%)	
		Total	19(73.1%)	7(26.9%)	26(100.0%)	
6th year	Shade Selection under Clinical light (Tooth # 11)	Correct	17(70.83%)	0(0.0%)	17(70.8%)	**0.004**
		Incorrect	4(16.66%)	3(12.5%)	7(29.2%)	
		Total	21(87.5%)	3(12.5%)	24(100.0%)	

A comparison of shade selection of posterior tooth # 36 under clinical and correcting light among clinical students revealed that a statistically significant difference was evident in shade selection under clinical and correcting light among 4th year students (*p* = 0.044). Around 11(42.3%) 5th year students selected shade correctly under clinical and correcting light, with a statistically significant association among them (*p* = 0.039). Moreover, 11(42.3%) 6th year students selected shade correctly under clinical and correcting light with a statistically insignificant association among them (*p* = 0.088), as shown in Table 3.

**Table 3:** The association of shade selection of tooth # 36 under clinical and correcting light with respect to the training level of students

Variables			Shade Selection under Correcting light (Tooth # 36)			
			Correct (n%)	Incorrect (n%)	Total (n%)	***P***-value
4th year	Shade Selection under Clinical light (Tooth # 36)	Correct	4(18.18%)	0(0.0%)	4(18.2%)	**0.044**
		Incorrect	8(36.36%)	10(45.45%)	18(81.8%)	
		Total	12(54.54%)	10(45.5%)	22(100.0%)	
5th year	Shade Selection under Clinical light (Tooth # 36)	Correct	11(42.3%)	2(7.7%)	13(50.0%)	**0.039**
		Incorrect	6(23.07%)	7(26.92%)	13(50.0%)	
		Total	17(65.4%)	9(34.6%)	26(100.0%)	
6th year	Shade Selection under Clinical light (Tooth # 36)	Correct	13(54.16%)	2(8.33%)	15(62.5%)	0.088
		Incorrect	5(20.83%)	4(16.66%)	9(37.5%)	
		Total	18(75.0%)	6(25.0%)	24(100.0%)	

The association between correct and incorrect shade selection under clinical and correcting light with respect to the training level of students revealed that most of the 6th year students selected the shade of the anterior tooth # 11 correctly under clinical light, although a statistically insignificant difference was evident among 4th, 5th, and 6th year clinical students (*p* = 0.122). Likewise, a statistically insignificant difference was evident among 4th, 5th, and 6th year clinical students in the shade selection of anterior tooth under correcting light (*p* = 0.374). As far as the shade selection of the posterior tooth is concerned, a statistically significant difference was observed under clinical light among all clinical students (*p* = 0.008). Whereas, an insignificant difference was found in shade selection between correct and incorrect shade selection of posterior tooth # 36 under correcting light among all clinical students (*p* = 0.347), as shown in Table 4.

**Table 4:** The association between correct and incorrect shade selection under clinical and correcting light with respect to the training level of students

Variables		Students training level			
		4th year	5th year	6th year	*p* value
Shade Selection under Clinical light (Tooth # 11)	Correct	9(40.9%)	14(53.8%)	17(70.8%)	0.122
	Incorrect	13(59.1%)	12(46.2%)	7(29.2%)	
Shade Selection under Correcting light (Tooth # 11)	Correct	16(72.7%)	19(73.1%)	21(87.5%)	0.374
	Incorrect	6(27.3%)	7(26.9%)	3(12.5%)	
Shade Selection under Clinical light (Tooth # 36)	Correct	4(18.2%)	13(50.0%)	15(62.5%)	**0.008**
	Incorrect	18(81.8%)	13(50.0%)	9(37.5%)	
Shade Selection under Correcting light (Tooth # 36)	Correct	12(54.5%)	17(65.4%)	18(75.0%)	0.347
	Incorrect	10(45.5%)	9(34.6%)	6(25.0%)	

## Discussion

Choosing the right shade of teeth for a prosthetic is a complicated procedure that necessitates a basic understanding of color and aesthetics. The dentist’s skill in selecting the right shade has an impact on both patient happiness and the efficacy of therapy. Many factors, such as background and light, can influence tooth color [25]. Therefore, the shade-matching ability will be enhanced when a reliable light source and suitable climatic conditions are used. While some studies support the use of traditional shade tabs, others take into account digital devices in order to achieve more accurate and precise measurements [26, 27]. Therefore, this study demonstrated the shade selection under clinical and correcting light by the clinical students.

Shade matching depends on the source of light. While it is true that natural sunshine is the ideal light source for matching shades, the quality of daylight is inconsistent, making it difficult to match shades at all times of the day. As a result, employing a reliable light source in conjunction with an appropriate ambient setting might enhance shade-matching performance [3]. This study demonstrated the shade-matching ability of posterior and anterior teeth by clinical male students under correcting and clinical lighting conditions.

The degree of education and training received in shade matching both show a strong correlation with shade matching accuracy. Previous research has shown that dental professionals need to participate in hands-on learning opportunities, continuing education initiatives, and further instruction in order to enhance their shade-matching ability [28]. In the present study, there was a significant difference (*p* < 0.05) between all shade tabs of the anterior tooth under clinical and correcting light among male 6th year students, indicating that students’ clinical experience with color matching improves the accuracy of shade selection. These findings were endorsed by another study, and their data should motivate dentists to actively participate in using their knowledge, explore precise training for shade matching, and incorporate color-corrected light devices into their dental skills [29]. Similar to the current investigation, another study found that students’ shade matching abilities under a color-correcting device improved shade selection in comparison to the traditional method under typical lighting circumstances [20].

Shade matching differs depending on the type of light source. Therefore, dental professionals must employ the appropriate light source in order to achieve the optimum shade and provide the patient with the best possible, aesthetically pleasing outcomes. An additional investigation was intended to determine how the clinical experiences of different dental students and interns affected the accuracy of shade matching. When compared to clinical light and daylight, that study showed a noticeably higher percentage of correct responses for identifying the proper shade under the correcting light source. According to that study, the experience of the students does not always influence the choice of shade [30]. These findings were corroborated by the present study and revealed that the majority of students chose the appropriate shade under correcting light rather than clinical light. The results of the present study support Nakhaei et al. (2013) and refute Gáspárik et al. (2014), who claimed that there is no distinction between the three light sources. A light source that resembled daylight was used to obtain the second-best shade-matching responses [3, 31]. These results were in contrast with Jabeen’s (2015) predilection for clinical light over daylight [32].

The present study revealed that the clinical experience of dental students plays a significant role in shade matching under lighting conditions. It was observed that most of the 6th year students selected the shade correctly under the correcting light. This was inconsistent with research by Helene et al., 2009 [15], in agreement with studies by Samra 2019 [33] and Jaju et al., 2010 [14], which indicate that professional experience is a major factor in shade matching. It is essential to advocate for and encourage students to practice this process more frequently, in addition to helping them make the right choices by increasing their knowledge, in order to prevent any issues in their future careers as dentists.

Shade matching is a significantly more complex process than it first appears, particularly when considering hue, value, and chroma. Nonetheless, training, exercise, and experience all contribute to an improvement in color perception with time [34]. Similarly, a study conducted in India, selected students from all academic years of the dental educational system demonstrated an increase in shade matching skill with their degree of dental training [35], which was in contrast with a related study conducted by Jaju et al. on the ability of dental students in the US to match shades. In the subsequent investigation, there was no significant correlation between the year of education and the ability to match tooth colors [14]. As far as the present study is concerned, it was revealed that level of the dental education showed an improvement in the shade selection of anterior and posterior teeth. Additionally, it was demonstrated that shade matching under correcting light was noticeably superior to that under clinical light.

Likewise, another study by Nakhaei et al. was performed to assess the impact of the type of shade guide and proficient skill on shade-matching outcomes. This study included 30 dental students, 30 general dentists, and 30 dental experts. When using the 3D shade guide, the shade-matching results did not differ among the three groups based on their level of experience [6]. This result is in line with research that found no relationship between expertise and shade matching [2, 36]. On the other hand, Dagg et al. found that expertise level affects shade matching under optimal lighting. Third-year dental students and other inexperienced observers did not obtain as accurate results as experienced practitioners, such as technicians and dentists. Nonetheless, there was no distinction between experienced and inexperienced observers when exposed to a mixture of fluorescent and natural light. Dagg et al.‘s study differed from the other one, possibly because they used a certain number of correct and incorrect matches as a gauge for shade-taking proficiency [37]. These findings were inconsistent with the present study that revealed the level of academic year had a significant influence on shade matching abilities. Additionally, shade selection under correcting light was better than in clinical lightning conditions.

The result of this study should be seen under certain limitations: multivariate analyses was not performed in this study as this research included only limited number of variables. Moreover, no female students participated owing to the fact that our institute only enrolled male students. Furthermore, because male students with less clinical experience were included in the study, its findings cannot be broadly applied.

It is recommended that more research be done to evaluate Dental Base Light (Tri-Shade, Zhengzhou, China) against alternative correcting lights and to carry out extensive randomized controlled clinical trials involving both genders in order to gain a better understanding of various computerized techniques perform in order to achieve successful shade matching. Additionally, there are spectrophotometers on the market that need further investigation in order to provide a thorough grasp of shade matching between the restoration and the tooth. At last, patients with other characteristics such as internal discoloration need further exploration.

## Conclusions

In summary, all students groups performed better in the shade selection under correcting light. Particularly, senior dental students possess a deeper understanding of the shade selection process, leading to enhanced clinician performance and more aesthetically pleasing patient outcomes.

## Data Availability

No datasets were generated or analysed during the current study.
